# Simulating an intra-fraction adaptive workflow to enable PTV margin reduction in MRIgART volumetric modulated arc therapy for prostate SBRT

**DOI:** 10.3389/fonc.2023.1325105

**Published:** 2024-01-08

**Authors:** Jeffrey Snyder, Blake Smith, Joel St. Aubin, Andrew Shepard, Daniel Hyer

**Affiliations:** Department of Radiation Oncology, University of Iowa Hospitals and Clinics, Iowa City, IA, United States

**Keywords:** VMAT (volumetric modulated arc therapy), intra-fraction, MRIgRT, adaptive, tracking, prostate SBRT treatment, MR-linac

## Abstract

**Purpose:**

This study simulates a novel prostate SBRT intra-fraction re-optimization workflow in MRIgART to account for prostate intra-fraction motion and evaluates the dosimetric benefit of reducing PTV margins.

**Materials and methods:**

VMAT prostate SBRT treatment plans were created for 10 patients using two different PTV margins, one with a 5 mm margin except 3 mm posteriorly (standard) and another using uniform 2 mm margins (reduced). All plans were prescribed to 36.25 Gy in 5 fractions and adapted onto each daily MRI dataset. An intra-fraction adaptive workflow was simulated for the reduced margin group by synchronizing the radiation delivery with target position from cine MRI imaging. Intra-fraction delivered dose was reconstructed and prostate DVH metrics were evaluated under three conditions for the reduced margin plans: Without motion compensation (no-adapt), with a single adapt prior to treatment (ATP), and lastly for intra-fraction re-optimization during delivery (intra). Bladder and rectum DVH metrics were compared between the standard and reduced margin plans.

**Results:**

As expected, rectum V18 Gy was reduced by 4.4 ± 3.9%, D1cc was reduced by 12.2 ± 6.8% (3.4 ± 2.3 Gy), while bladder reductions were 7.8 ± 5.6% for V18 Gy, and 9.6 ± 7.3% (3.4 ± 2.5 Gy) for D1cc for the reduced margin reference plans compared to the standard PTV margin. For the intrafraction replanning approach, average intra-fraction optimization times were 40.0 ± 2.9 seconds, less than the time to deliver one of the four VMAT arcs (104.4 ± 9.3 seconds) used for treatment delivery. When accounting for intra-fraction motion, prostate V36.25 Gy was on average 96.5 ± 4.0%, 99.1 ± 1.3%, and 99.6 ± 0.4 for the non-adapt, ATP, and intra-adapt groups, respectively. The minimum dose received by the prostate was less than 95% of the prescription dose in 84%, 36%, and 10% of fractions, for the non-adapt, ATP, and intra-adapt groups, respectively.

**Conclusions:**

Intra-fraction re-optimization improves prostate coverage, specifically the minimum dose to the prostate, and enables PTV margin reduction and subsequent OAR sparing. Fast re-optimizations enable uninterrupted treatment delivery.

## 1 Introduction

Prostate cancer is the second most prevalent type of cancer among men in the United States with an estimated 288,300 newly diagnosed cases in 2023 (1). Radiation therapy has an important role in the treatment of prostate cancer with an estimated 60% of patients requiring radiation therapy at some point over the course of their disease (2, 3). Treatments have proven to be very effective as evidenced by the 99% overall survival rate at 10 years for patients who have localized disease and who are diagnosed at early stage with low to intermediate risk of recurrence (4).

Studies have shown that the prostate has a low alpha/beta ratio of approximately 1.5 while nearby critical organs at risk (OAR) such as the bladder and rectum have alpha over beta ratios in the range of 3 -5 Gy for late toxic effects (5–9). These radiobiological factors indicate that the prostate is sensitive to high dose per fraction treatments. This makes prostate SBRT an attractive treatment option which has now become an increasingly used treatment method (10). Clinical trials comparing survival and toxicity profiles have shown non-inferiority for SBRT as compared to conventional fractionation in the treatment of prostate cancer (11–13). SBRT also reduces the number of fractions which improves patient satisfaction and is a more cost-effective treatment as compared to conventional fractionation (5, 14–16). While these are positive factors and the toxicity rate for prostate SBRT is generally considered acceptable, side effects remain. Alongi et al. reported a 40% incidence of grade 2 Genito-urinary (GU) toxicities while Kishan et al. reported a 10% incidence of grade 2 or greater gastrointestinal (GI) toxicity (17, 18). With a high overall survival rate, a focus on developing treatment strategies which reduce side effects should remain a priority.

One method which will better spare OAR’s and potentially reduce treatment related side effects is the reduction of PTV margins (19). Reducing PTV margins poses challenges as the prostate exhibits both inter and intra-fraction motion caused by bladder and rectal filling, bowel movement, and skeletal muscular motion (20–22). Therefore, caution should be employed when implementing margin reductions because advanced imaging technologies and strategies may be required to prevent underdosage of the target (23, 24). Prostate PTV margins ranging from 2 mm to greater than 10 mm have been reported in the literature and this variation often coincides with the type of pretreatment imaging used and whether intra-fraction adaptions are applied (25). Most commonly, PTV margins fall within the range of 4 to 6 mm (22, 26–28). Keizer et al. found that PTV margin reduction below 4 mm would require intra-fraction monitoring and correction (29). Common intra-fraction monitoring techniques used in prostate radiotherapy include the use of triggered planar imaging of implanted fiducials or through electromagnetic tracking of implanted beacons (30–33). While these methods aid in monitoring and correcting intra-fraction motion, they have drawbacks including an invasive seed implantation and an extra appointment for the patient (34). Additionally, triggered imaging methodologies add additional non-target specific ionizing radiation, and electromagnetic beacons cause artifacts limiting the use of MRI in delineation of the prostate (31, 35). Lastly, these intra-fraction correction methods can add additional treatment time. Gorovets et al. reported a maximum fractional treatment time of 45 minutes when monitoring with kV/MV imaging on a standard linear accelerator and implementing a 2 mm correction threshold (36). Furthermore, Kisivan et al. reported that 29% of treatment fractions would require greater than 1 intra-fraction intervention when using a 3 mm motion threshold (37).

MRI guided adaptive radiotherapy (MRIgART) has emerged as a promising technique for treating prostate cancer. MRIgART enables real time cine imaging and target tracking without additional ionizing radiation or fiducial markers (38, 39). It is estimated that one third of prostate patients require adaptive radiotherapy which can be applied online with MRIgART (40). The MIRAGE trial compared non-adaptive MRI linac based treatments using reduced margins versus treatments delivered with conventional linacs and standard margins, finding that MRI guided radiotherapy reduced GU and GI toxicities (18). Additionally, in a meta-analysis of 29 prospective studies, Leeman et al. found that MRI guided radiotherapy reduced urinary side effects by 44% and bowel side effects by 60% as compared to conventional CT guided treatment methods with implanted fiducials (41). Combining reduced PTV margins and online adaptive re-planning may further reduce treatment related toxicities. However, long treatment session times associated with MRIgART and intra-fraction motion limit the extent to which PTV margins can be reduced without intra-fraction compensation (29, 42, 43). MRIgART with daily re-planning commonly uses prostate PTV margins ranging between 3 and 5 mm (44–48) with 5 mm in all directions except for 3 mm in the posterior direction remaining a standard (43, 49–51). While techniques such as gating and/or baseline shift corrections can be used in conjunction with reduced PTV margins, this will add additional time to already long treatment sessions and therefore is impractical for some patients (42, 43). This may be especially impactful on systems such as the Elekta Unity which does not support couch movement during treatment and thus, users must wait for baseline shift plans to be re-optimized or re-calculated prior to resuming treatment if target excursions occur (52, 53).

Recently, the use of 2D cine MRI imaging coupled with target tracking has been used to reconstruct the fractional dose delivered to the target for prostate and seminal vesicle treatments (29, 48, 54). Additionally, the feasibility of VMAT treatment delivery techniques have been shown which reduce delivery time as compared to step-and-shoot IMRT, the current standard for all MRI-linacs (55, 56). This study builds on these earlier works by simulating a novel intra-fraction MRIgART workflow that combines cine MRI target tracking and VMAT delivery to enable intra-fraction dose re-optimization without causing delays in treatment. The efficacy of the workflow to maintain adequate prostate coverage with 2 mm PTV margins while accounting for intra-fraction motion was also evaluated.

## 2 Materials and methods

### 2.1 Patient selection and reference planning

Ten prostate cancer patients previously treated on our Elekta Unity MRI linac were enrolled in this retrospective planning study. All patients provided informed prospective consent to use their treatment images and this study was conducted in accordance with the International Council for Harmonization ICH E6 (R2) Good Clinical Practice as adopted by the United States FDA, which aligns with the principles of Helsinki. Each patient received a simulation CT scan with 2 mm slice thickness as well as a diagnostic T2 MRI image to aid in the delineation of the prostate. In this study, all patients were simulated to be low risk prostate cancer patients without seminal vesicle or nodal involvement. A SpaceOAR hydrogel spacer was placed for all patients except for patient 7 who declined. The SpaceOAR increases the separation between the prostate and rectum and aids in the reduction of rectal dose (57). The gross tumor volume consisted of the prostate and no additional expansion was used for the CTV (GTV = CTV). A research Monaco treatment planning system (version 6.09.00) was used to generate reference MRIgART VMAT treatment plans using two separate PTV margins. The standard PTV margin group consisted of a 5 mm expansion of the CTV in all directions except for 3 mm posteriorly, while the reduced PTV margin group used a uniform 2 mm expansion of the CTV. A previously commissioned clinical beam model was used to generate all plans within this study (52). For this study, all plans were optimized to deliver 36.25 Gy in 5 treatment sessions and normalized for 95% PTV coverage. OAR dose limits for planning followed our institutional standard which is based off of the criteria published by the PACE B clinical trial and Tanaka et al. (26, 58).

### 2.2 Cine MRI target tracking

Cine MRI images were acquired during radiation delivery for each fraction and all patients enrolled in this study. The cine MRI consisted of a balanced T1/T2 fast spin echo (TE 3.8 ms, TR 1.92 ms, flip angle: 40 degrees) imaging sequence which comes standard on the Unity system (59). The cine MRI sequence had temporal resolution of 200 msec with coronal and sagittal images being acquired in an interleaved fashion. The pixel size of the cine MRI images was 1.13 mm with a 5 mm slice thickness (60).

**Figure 1:**
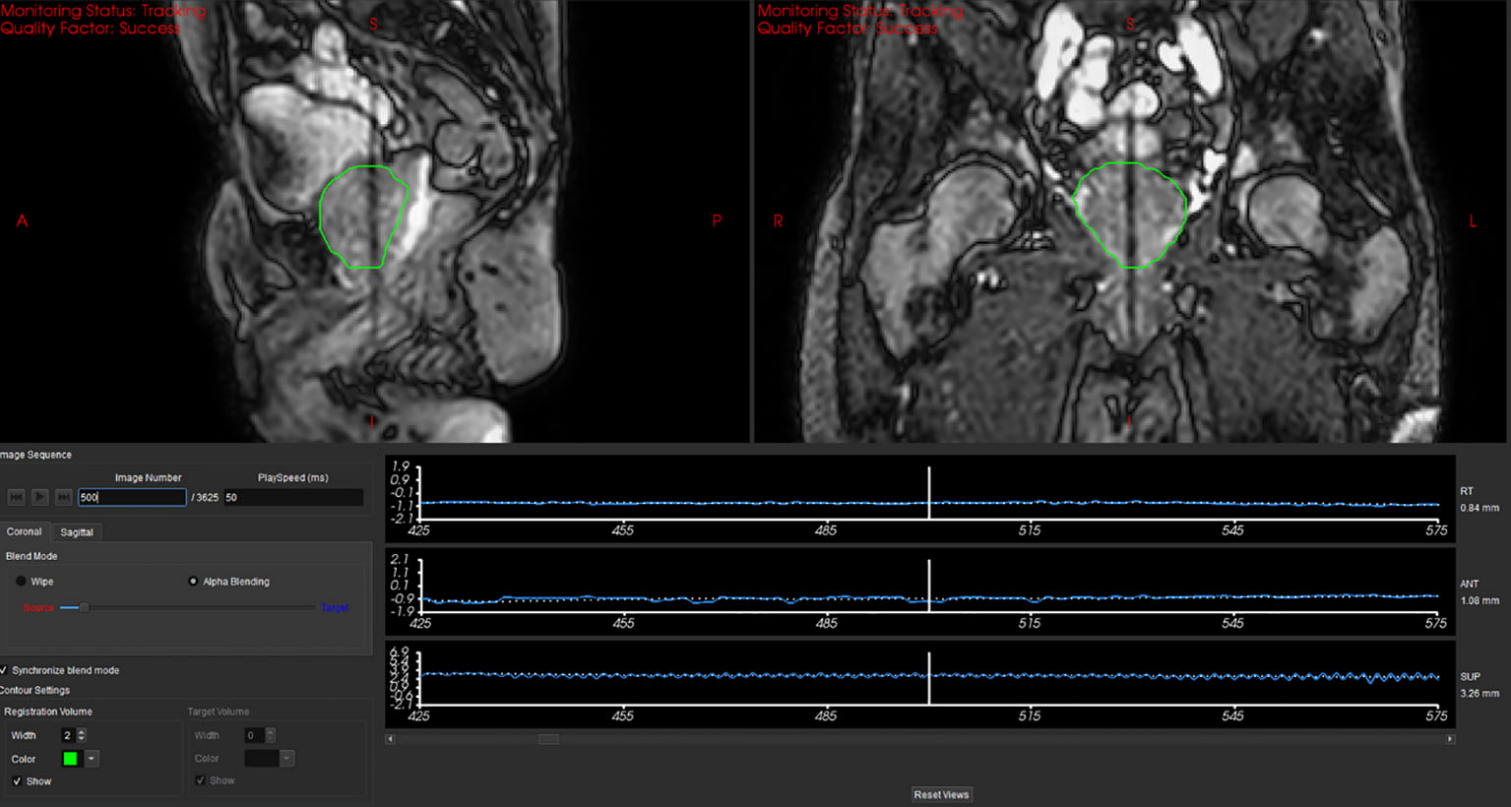
Screen Capture depicting the motion tracking software interface. The green contour represents the position of the prostate as identified by the tracking algorithm in the live cine MRI in a sagittal and coronal plane. The bottom of the image depicts the tracked position of the prostate in the right/left, ant/post, and sup/inf positions on the current frame as well as history of previous tracked positions.

A preclinical motion management research package (MMRP) was used to retrospectively analyze and track the position of the prostate in each frame of the cine MRI acquisitions. Details of the target tracking algorithm have been previously published (39, 60, 61). Briefly, the algorithm begins by using 2D-3D template matching. In this stage the first 60 cine MRI images (30 sagittal and 30 coronal) are used to generate a single average 2D coronal and 2D sagittal image which serve as the template images. These template images are then registered to a coronal and sagittal slice which is extracted from the 3D MRI dataset at the centroid location of the prostate. This registration can be manually edited by the user if necessary. In the next stage, live cine MRI images are automatically registered to the average 2D template images generated during the first stage (2D-2D). The total offset of the prostate in each cine frame is then equal to the summation of the initial 2D-3D registration plus the registration value of the 2D-2D matching. An example of the target tracking interface and live prostate identification by the MMRP algorithm is shown in **Figure 1**.

### 2.3 Online adaptive planning procedure incorporating target tracking

Both the standard PTV margin group and the reduced PTV margin group began with a standard adapt-to-shape workflow in the online environment (62). Briefly, a 2 min T2 weighted MRI image was acquired at the beginning of each treatment fraction. The prostate and OARs were contoured on each daily MRI dataset (50 fractions in total). Once contouring was completed a full optimization of the VMAT plans was performed using a pseudo gradient descent and optimal fluence levels optimizer within the research Monaco TPS (43). The standard margin plans were optimized with 1% statistical uncertainty per plan, 3 mm dose grid, and were scaled for 95% PTV coverage. Each reference plan was generated using two counterclockwise treatment fields with the first beam going from 179° to 21° and the second VMAT arc treating from 5° to 180°. This is equivalent to a single full VMAT arc, but without treating through the cryostat pipe of the MR linac (59). All plans used a minimum segment width of 0.75 cm and a maximum of 90 control points per beam. For the standard PTV margin group no further adaptation was performed and this group serves as a control for comparison with the intra-fraction re-optimization reduced PTV margin group as described below.

**Figure 2:**
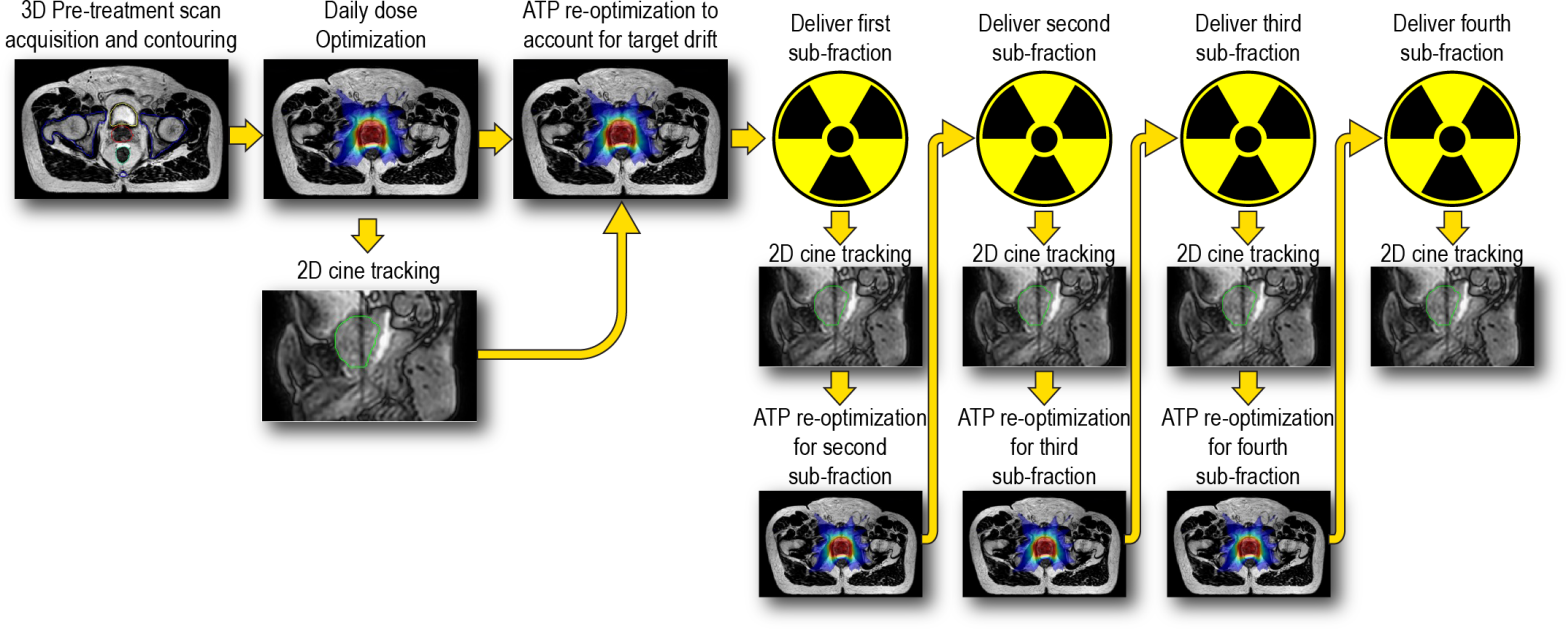
Overview of intra-fraction adaptive workflow incorporating near real time optimization in parallel with dose delivery. Cine MRI imaging starts at the onset of the intital dose optimizations and remains running throughout the entirety of the treatment session.

For the reduced margin group, a novel workflow was performed which consisted of breaking the online treatment session into 4 sequential sub-fractions per treatment session. Each sub-fraction was planned to deliver a uniform 25% (1.8125 Gy) of the total treatment session dose. Essentially four subsequent VMAT arcs were used to deliver the total SBRT dose per fraction of 7.25 Gy. In this workflow, a re-optimization is performed in parallel with the delivery of each arc such that a new fully re-optimized plan that includes any intrafraction drift of the target is ready for delivery at the immediate conclusion of the current arc. Thus, intra-fraction adaptive plans will be delivered without any pauses in treatment delivery. **Figure 2** shows a diagram depicting this workflow.

The aim of this workflow is to compensate for intra-fraction motion of the prostate and maintain prostate coverage with reduced PTV margins. To accomplish this goal, the position of the prostate, as identified by the tracking algorithm and cine MRI, would need to be synchronized with the radiation treatment delivery. Immediately after the original ATS optimization, an adapt-to-position (ATP) re-optimization technique would applied which shifts the MLC’s based on the current position of the prostate and re-weights each IMRT segment to reproduce the dose distribution of the daily ATS plan (ATP “optimize weights”) (62). This step would account for intra-fraction motion of the prostate which occurred during the re-contouring and original optimization timeframe. The first VMAT treatment arc would then be delivered following the ATP re-optimization. The Monaco treatment planning system reports the delivery time of each treatment beam and in this workflow a re-optimization would be triggered 65 seconds prior to the completion of the active VMAT arc. 65 seconds was chosen in this work and represents a conservative value to ensure that the plan would be finished optimizing and exporting prior to the completion of the active arc. Thus, the second arc begins delivery with a newly adapted sub-fraction plan which is corrected for intra-fraction motion. This process repeats until all four VMAT sub-fractions have been delivered. All plans using the reduced PTV margins were calculated with 2% statistical uncertainty per sub-fraction, 3 mm dose grid, and were scaled to 95% PTV coverage. The use of 2% statistical uncertainty speeds up optimization and because 4 repeated Monte Carlo calculations will be done, the final composite dose from all 4 fractions will have an inherent 1% statistical uncertainty per plan, which is commonly reported in clinical use. In addition, the use of 2% statistical uncertainty has been shown to have negligible dosimetric impact as compared to plans that use 1% statistical uncertainty per plan (63). The optimization and the reported delivery times for each sub-fraction were recorded. In total, an additional 200 intra-fractional adaptive plans were created in this study.

### 2.4 Sub-fraction workflow composite dose calculation and OAR comparison

Sub-fraction doses from each treatment session were rigidly registered back onto the daily MRI dataset. These sub-fraction doses were then summed to generate a composite daily fractional dose on the daily MRI dataset which was equal to 7.25 Gy per fraction, matching the standard margin dose and fractionation scheme. OAR doses from the composite reduced margin group were compared against the daily adapted ATS plans for the standard margin group. OAR dose metrics were evaluated at critical constraint values as specified by the PACE B trial (58). OAR metrics evaluated include V18 Gy, D1cc, D5cc for bladder and V18 Gy, and D1cc for the rectum. The tracking algorithm used in this study tracked the prostate only and is not capable of accounting for deformations like those commonly seen for the bladder and rectum. For these reasons, all reported OAR doses in this this study are as calculated on the daily MRI dataset and do not account for intra-fractional motion. Differences between the standard and reduced PTV margin groups were evaluated for statistical significance for each individual DVH metric using a two sided t-test and 0.05 significance level.

### 2.5 Intra-fraction prostate motion and dose reconstruction

Sub-fraction doses were first rigidly registered onto the daily MRI dataset. Next, intra-fraction dose was accumulated following the methodology provided by Snyder et al. (39). Briefly, the prostate position as a function of time defined from the cine MRI images and target tracking was synchronized with the radiation therapy treatment delivery. The DICOM coordinates from each of the eight treatment arcs were shifted to account for the prostates average position during the delivery of that beam. In this scenario, the isocenter position of each beam was shifted to represent the effective motion of the beam with respect to the fixed reference of the prostate on the daily MRI image (39). The shifted beam doses were then summed and composite doses were viewed within Velocity (Varian Medical Systems Inc., Palo Alter, CA, version 3.2.1).

**Figure 3:**
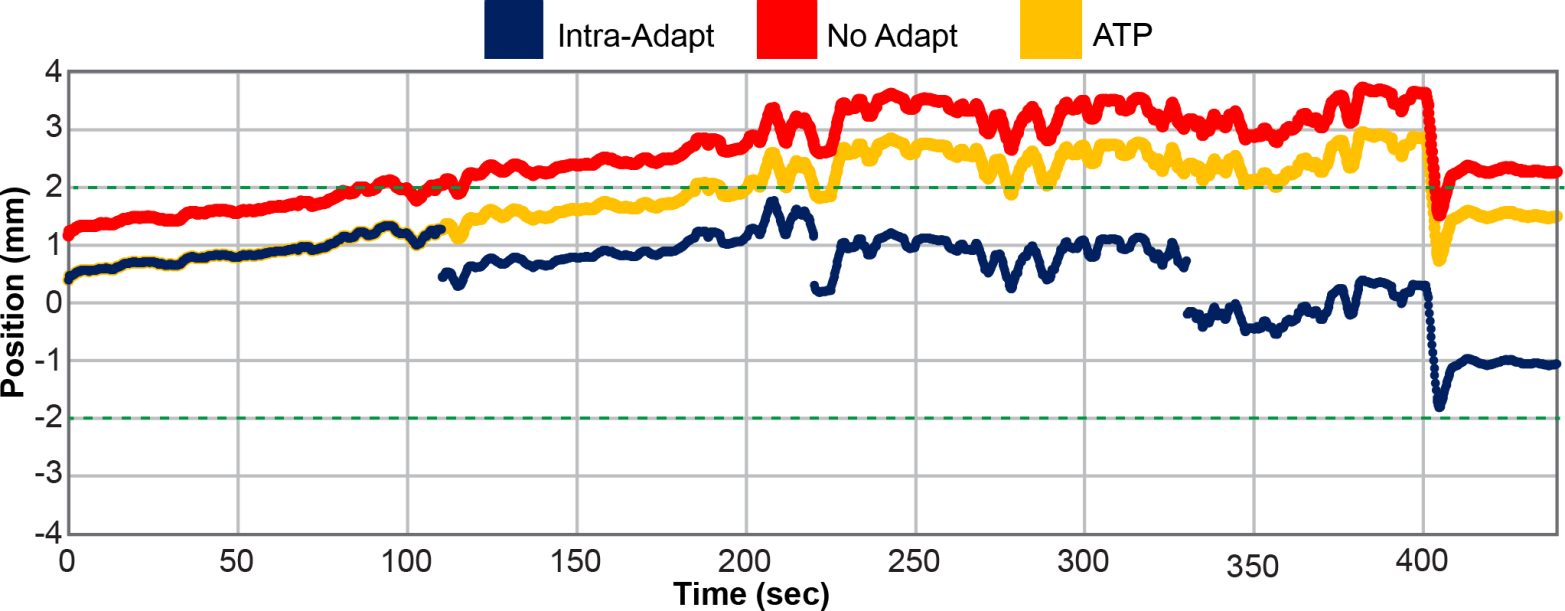
Prostate motion trace for patient 5 fraction number 4 in the Anterior (-) and Posterior (+) direction. The position of the prostate is plotted as a function of time for the unaltered no adapt motion trace (red), motion trace where adaption was only applied prior the start of treatment (ATP) (yellow), and for the full intra-fraction adaptive workflow (intra-adapt) (navy blue). The green dashed horizontal line depicts the uniform 2 mm PTV margin expansion from the originally planned prostate position.

To evaluate the utility of the intra-fraction adaptive workflow, the motion trace from the cine MRI images were simulated under three scenarios, as depicted in **Figure 3**. In the first scenario, the motion trace was unaltered from the clinical delivery and thus represents no adaptation. This case reflects motion which occurs throughout the adaptive process time including contouring and ATS optimization. This is referred to as the “no-adapt” group. In the second scenario, the motion trace was modified to reflect performing an ATP virtual couch shift immediately prior to treatment. This strategy has been a previously reported workflow for MRIgART (29). In this scenario, no other intra-fraction corrections are considered and no further modifications were made to the prostate motion trace. This scenario is referred to as “ATP”. The last scenario represents the full intra-fraction re-optimization technique proposed in this study. This scenario begins like the “ATP” group, but the prostate motion trace is further modified to reflect each time point when a new intra-fraction re-optimized plan were to begin. Therefore, this scenario includes four updates to the original prostate motion trace which correspond to time points at which the newly adapted plans begin. This scenario is referred to as “intra-adapt”. Of note, the first 25% of the treatment session motion traces are the same for the “ATP” and “intra-adapt” groups.

The effect of intra-fraction motion on the dose received by the prostate was compared for each scenario. Coverage metrics include prostate V36.25 Gy, minimum dose received by the prostate, and PTV V36.25 Gy. Statistical significance was assessed using a one-way ANOVA and a Bonferroni *post hoc* analysis.

## 3 Results

### 3.1 OAR dosimetric comparison of reduced and standard PTV margins

**Figure 4:**
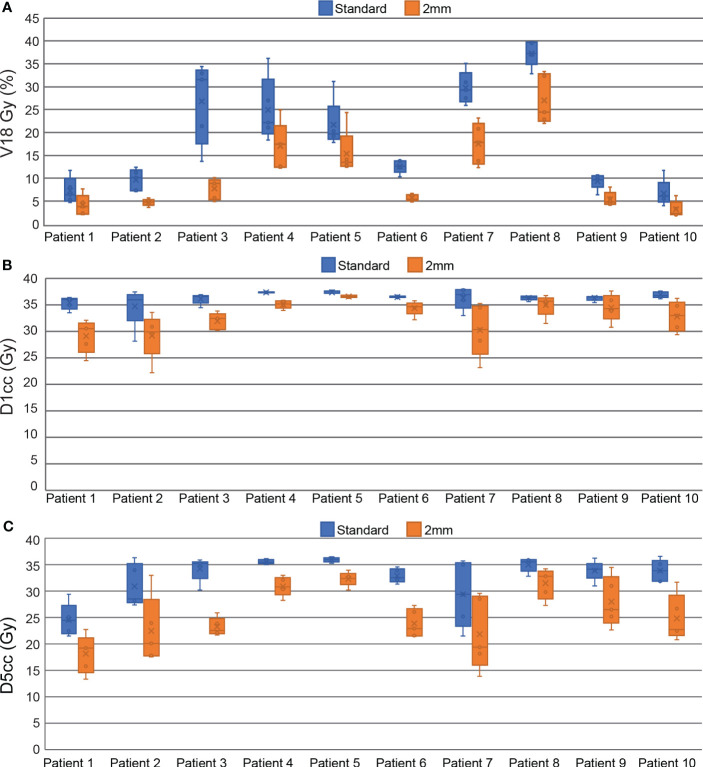
Box and Whisker plots comparing standard and reduced margin plans at key bladder dosimetric constraint values including V18 Gy **(A)**, D1cc **(B)**, and D5cc **(C)**.

**Figure 5:**
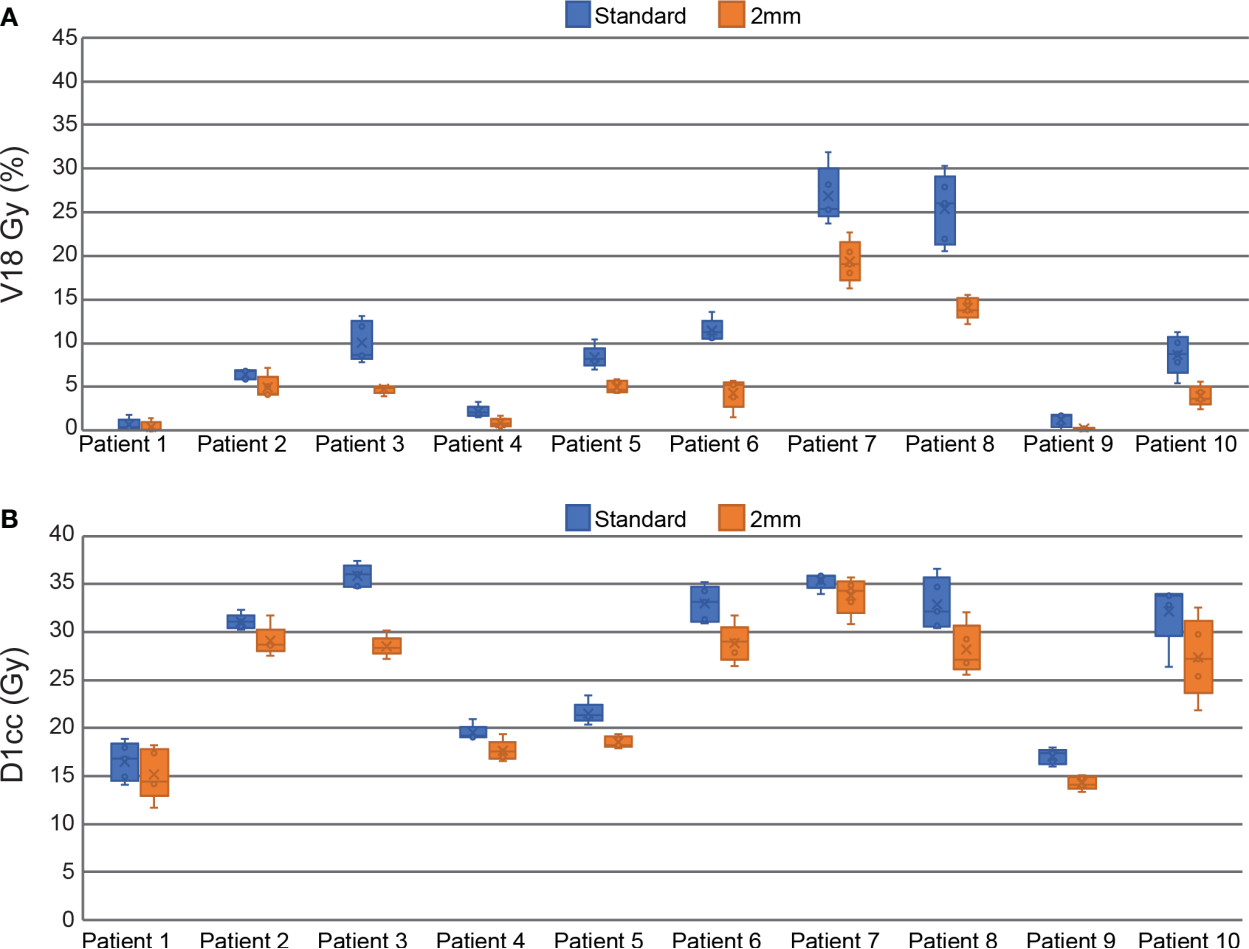
Box and Whisker plots comparing standard and reduced margin plans at key rectum dosimetric constraint values including V18 Gy **(A)**, and D1cc **(B)**.

All plans in this study meet the mandatory dosimetric constraints for prostate SBRT as defined by our institutional standards. This includes all reference plans, all ATS plans in the standard PTV margin group, and all the composite sub-fraction workflow adapted plans in the reduced margin group. The average separation between the prostate and rectum created by the SpaceOAR was 0.9 ± 0.4 cm (range 0.3 – 1.6 cm). The intra-fraction composite plans in the reduced margin group had statistically significant reductions in all DVH points analyzed in this study including bladder V18 Gy, D1cc, D5cc, and rectum V18 Gy and D1cc. Bladder DVH metrics were reduced by 7.8 ± 5.6% for V18 Gy (p<0.001), 9.6 ± 7.3% (3.4 ± 2.5 Gy) for D1cc (p<0.001), and 21.9 ± 10.3% (6.9 ± 3.0 Gy) for D5cc (p<0.001). Rectum V18 Gy was reduced by 4.4 ± 3.9% (p<0.001) and D1cc was reduced by 12.2 ± 6.8% (3.4 ± 2.3 Gy) (p<0.001). All of these DVH metrics are based on OAR contours generated on the daily MRI datasets and do not account for intra-fraction motion. Per patient bladder DVH metrics are displayed in the box and whisker plot in **Figure 4** and per patient rectum metrics are shown in **Figure 5**.

### 3.2 Intra-fraction re-optimization timing and prostate motion trace

The average time to complete intra-fraction re-optimizations among all 200 sub-fractions was 40.0 ± 2.9 seconds while the average time to deliver a single sub-fraction was estimated to be 104.4 ± 9.3 seconds. With this approach, every re-optimization would be completed prior to the completion of the sub-fraction currently being delivered. Thus, the proposed intra-fraction optimization workflow would not cause any interruptions in treatment delivery. The average per patient re-optimization times among all 20 fractions and sub-fraction delivery times are shown in **Table 1**.

**Table 1:** Intra-fraction workflow timing.

	Intra-fraction optimization time (sec)	Estimated sub-fraction delivery time (sec)	total treatment delivery time (sec)
Patient 1	41.8 ± 2.7	111.6 ± 17.0	446.3 ± 68.0
Patient 2	41.6 ± 2.8	108.4 ± 5.1	433.5 ± 20.6
Patient 3	39.7 ± 1.7	104.4 ± 4.4	417.6 ± 17.6
Patient 4	37.9 ± 1.8	104.0 ± 4.5	416.0 ± 18.1
Patient 5	40.0 ± 1.3	102.3 0± 4.6	409.2 ± 18.3
Patient 6	35.3 ± 2.4	110.6 0± 11.1	442.3 ± 44.4
Patient 7	40.0 ± 1.8	95.3 0± 3.9	381.4 ± 15.7
Patient 8	42.1 ± 1.4	93.7 0± 2.8	374.6 ± 11.2
Patient 9	43.2 ± 1.2	101.1 0± 3.5	404.4 ± 14.0
Patient 10	38.9 ± 1.3	113.1 0± 6.2	452.6 ± 24.9

**Figure 3** shows an example of a single fraction motion trace for patient 5 in the anterior (–) and posterior (+) directions. The motion trace with no adaptions reaches an excursion of nearly 4 mm with respect to the originally planned prostate position, while the ATP group had a maximum excursion of 3 mm and the intra-adapt group had an excursion of less than 2 mm. This is a representative fraction from this study. The no-adapt motion trace does not start at 0 mm at time point zero because of prostate drift that occurred during the adaptive planning process. The Intra-adapt and ATP groups also do not start at time point zero because the adaptions do not instantaneously take effect. Optimization is initiated and during the re-planning process the prostate position can drift before the new sub-fraction is ready for delivery. **Figure 3** highlights the benefits of using the intra-adaptive workflow to reduce prostate excursions from the planned position during treatment delivery.

**Figure 6:**
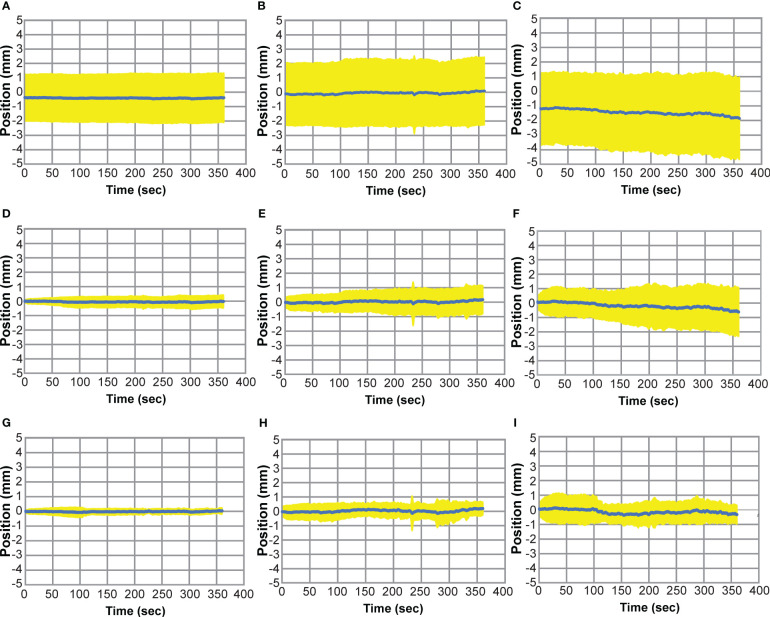
Prostate Motion Traces where the average prostate position (blue) is shown as a function of time over all treatment fractions and the standard deviation at each time point is shown in yellow. Motion traces for the non-adapt group are shown for left (+)/right (-) **(A)**, ant (-)/post (+) **(B)**, and sup (+)/inf (-) **(C)**. Motion traces for the ATP group are shown for left/right **(D)**, sup/inf **(E)**, and ant/post **(F)**. Motion traces for the intra-adapt group are shown for left/right **(G)**, sup/inf **(H)**, and ant/post **(I)**.

**Figure 6** shows the composite motion traces from all 50 fractions of the no-adapt (A-C), ATP (D-F), and intra-adapt (G-I) groups. The average position of the prostate among all fractions for each time point is depicted by the blue line and the standard deviation at each time point is shown by the yellow color wash. During planning and treatment delivery the average position of the prostate had a systematic drift in the inferior direction as noted by the negative values in **Figure 6C** and a small systematic drift in the posterior direction (**Figure 6B**). The standard deviation in the position of the target at the onset of treatment was greatest for the no adapt-group, attributed to the fact that the motion was not accounted for with an ATP plan directly prior to treatment. At the final time point in in **Figure 6** (treatment completion), the magnitude and standard deviation of the prostate as compared to the initial planned position was -0.39 ± 1.72 mm (Left/Right), 0.09 ± 1.72 mm (Ant/Post), and -1.86 ± 2.82 mm (sup/inf) for the no-adapt group, 0.03 ± 0.44 mm (Left/Right), 0.17 ± 0.98 mm (Ant/Post), and -0.62 ± 1.68 mm (sup/inf) for the ATP group, and -0.02 ± 0.21 mm (Left/Right), 0.19 ± 0.46 mm (Ant/Post), and -0.33 ± 0.65 mm (sup/inf) for the intra-adapt group. This shows that the intra-adapt workflow effectively corrects the systematic prostate drift observed in the no-adapt group. The intra-adapt workflow also has the smallest variability in prostate position at the end of each treatment session delivery, as indicated by the intra-adapt group having the smallest standard deviation in each of the three principal motion directions.

### 3.3 Intra-fraction reconstructed prostate and PTV dose coverage

The delivered dose of the day was reconstructed while accounting for intra-fraction motion of the prostate. The percentage of the prostate covered by the prescription isodose line (V36.25 Gy) was on average 96.5 ± 4.0%, 99.1 ± 1.3%, and 99.6 ± 0.4 for the non-adapt, ATP, and intra-adapt groups, respectively. The minimum dose received by the prostate was on average 31.5 ± 4.7 Gy, 34.4 ± 1.7 Gy and 35.1 ± 0.8 Gy for the non-adapt, ATP, and intra-adapt groups, respectively. The minimum dose received by the prostate was less than 95% of the prescription dose in 84%, 36%, and 10% of fractions, for the non-adapt, ATP, and intra-adapt groups, respectively. The PTV V36.25 Gy was on average 90.0 ± 4.7%, 94.6 ± 2.4%, and 95.6 ± 1.2 for the non-adapt, ATP, and intra-adapt groups, respectively. For all metrics evaluated, the intra-adapt group had the smallest variability, thus having smaller deviations with respect to prescribed dose on the static daily MRI image.

**Figure 7:**
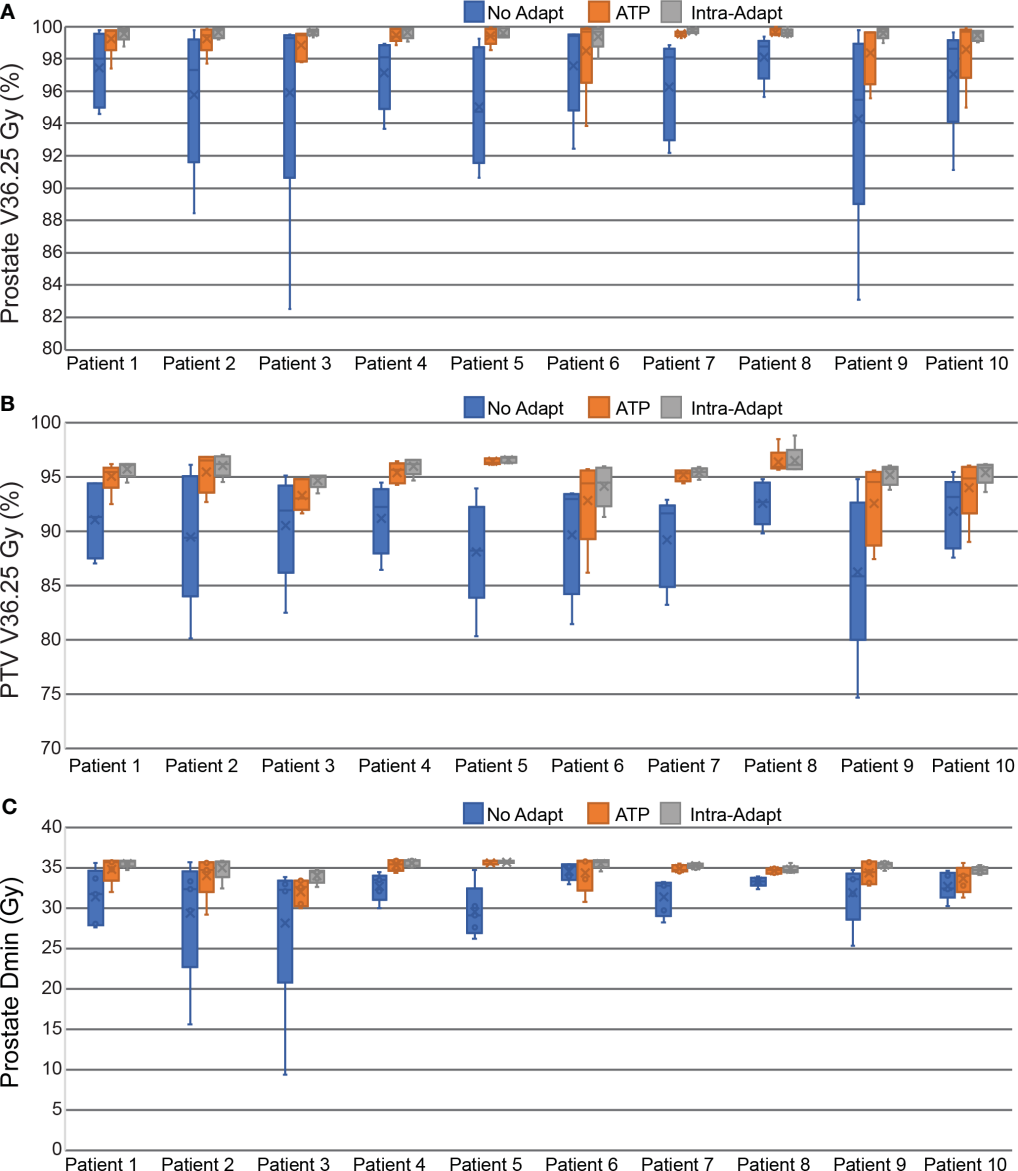
Box and Whisker plots measuring the dosimetric impact of prostate intra-fraction motion during treatment delivery. Dose and coverage statistics are shown for the prostate V36.25 Gy **(A)**, PTV V36.25 Gy **(B)**, and the minimum dose received by the prostate **(C)**.

**Figure 7** displays the per patient intra-fraction accumulated DVH statistics for prostate V36.25 Gy (A), PTV V36.25 Gy (B), and the minimum dose received by the prostate (C). The box and whisker plot represents the statistics from each of the 5 fractions for each patient.

## 4 Discussions

Reducing margins in prostate radiotherapy remains a challenge due to intra-fraction motion. MRIgART can track targets without using invasive fiducial implants or additional ionizing radiation which is advantageous over tracking methodologies on conventional linear accelerators. However, the Elekta Unity MR linac design does not support couch motion (52) and therefore intra-fraction baseline shift corrections require a re-calculation and/or re-optimization. Reduced PTV margins will necessitate more frequent intra-fraction adaptions and re-optimizations. This may significantly decrease the beam-on duty cycle and add extra time to the treatment delivery. MRIgART already has a limitation of long treatment session times, therefore intra-fraction adaptive techniques which add extra treatment time are not desirable. This study overcomes that limitation by simulating the feasibility of an intra-fraction re-optimization workflow based on the position of the target extracted from the 2D cine images acquired during treatment delivery that can be performed in parallel with radiation delivery such that no beam pauses are required.

The commonly used Van Herk PTV margin methodology is designed to ensure that 90% of patients receive a minimum CTV dose of at least 95% of the prescription dose (64). In this study we found that this minimum dose metric was not met in 84%, 36%, and 10% of fractions when using the no-adapt, ATP, and intra-adapt methodologies with a 2 mm margin. Thus, our study has shown that 2 mm margins are not feasible for the no-adapt and ATP methodologies. This is similar to findings presented by Keizer et al. who reported that PTV margin reductions below 4 mm would require intra-fraction adaptions for prostate MRIgART treatments (29). Previously reported MRIgART motion management studies have focused on step-and-shoot IMRT deliveries while this study simulated a VMAT delivery technique. Willigenburg et al. reported prostate step-and-shoot delivery times of 11.0 minutes (65) while this study had an average total treatment session delivery time of 7.0 minutes. Despite the shorter treatment times afforded by VMAT, 2 mm margins were still not feasible without further intra-fraction corrections. However, by using the intra-adapt workflow, 2 mm PTV margins are feasible based on the Van Herk definition. While the intra-adapt workflow does add additional complexity, the ability to reduce PTV margins highlights its utility.

PTV margin reductions have been previously shown to provide superior OAR sparing in prostate SBRT (23). This study found statistically significant reductions for all DVH metrics in the reduced margin group as compared to the standard PTV margin group. This will likely lead to decreased GU and GI toxicities as demonstrated by the MIRAGE trial (18). Improved OAR sparing may be even more critical in studies which attempt to perform even more extreme hypofractionation and treat the prostate in one to two fractional doses (66, 67). Reducing margins to better spare OARs is also of paramount importance in SBRT re-irradiation in the recurrent prostate setting (68).

Prior studies have shown that intra-fraction motion can degrade OAR sparing in MRIgRT by comparing pre and post image MRI scans. Brennan et al. found that urethral sparing was achieved in 92% of fractions as defined in the pre MRI scan but that number degraded to 66% of fractions meeting the same dosimetric constraint on the post MRI image (69). Similarly Dang et al. found that intra-fraction motion caused a statistically significant increase in the bladder D5cc dose (70). While the primary focus of this study was to reconstruct the daily delivered dose to the prostate, it is our hypothesis that the proposed intra-fraction re-planning workflow will also reduce the degradation in OAR sparing caused by intra-fraction motion. Future studies should be designed to test this which use intra-fraction imaging to specifically track these OARs and which are capable of tracking non-rigid structures.

Recently, a sub-fraction workflow in MRIgART was proposed by Willigenburg et al. (65). In this study, a 3D MRI image was acquired in parallel with IMRT treatment delivery and an ATP adaptation was performed based on the target position on the intra-fraction 3D MRI scan while the first half of treatment was being delivered. The novelty of our work comes from the increased efficiency of using cine MRI imaging to perform multiple re-optimizations within one treatment session while allowing continuous live tracking of the prostate such that the beam can be paused should any large sudden motions occur. The proposed workflow incorporates VMAT and enables uninterrupted treatment from arc to arc. In this work sub-fractions took on average 94.5 seconds to deliver while optimization was performed in 40.1 seconds on average. This differential highlights that an even larger number of sub-fractions could be implemented for patients who experience consistently large intrafraction motion without causing treatment pauses while waiting for the new treatment plan. The ability to efficiently perform multiple sub-fraction adaptations showed the feasibility of using 2 mm margins while Wiligenburg’s sub-fraction workflow, while beneficial, still required PTV margins up to 2.6 mm. Since each VMAT arc delivers a uniform 25% of the treatment session dose, no intra-fraction deformable dose accumulation was needed. This is a similar strategy to that employed by Willigneburg (65) and simplifies the online re-optimization.

While this work focused on using SBRT to treat the entire prostate it can also be extended to simultaneous integrated boost techniques in cases where the GTV is identifiable. Currently, the balanced cine MRI imaging sequence would not provide adequate contrast to track the GTV directly, but this intrafraction sub-fraction workflow could be employed if the prostate and GTV are assumed to move as a rigid body throughout the course of each fraction. Cine MRI imaging sequences with different weightings may enable direct tracking of the gross tumor within the prostate in the future.

One of the limitations of this study is the relatively small number of patients evaluated. However, this study was able to achieve statistically significant differences in OAR sparing and target coverage between each adaptive strategy and PTV margin group. While the number of patients was small, a total of 320 plans were evaluated between reference and adaptive plans.

## 5 Conclusion

When using reduced PTV margins, intra-fraction adaptation is needed to account for prostate motion and to prevent underdosage to the prostate and PTV. Reduced PTV margins provide superior OAR sparing as compared to standard margin expansions. The intra-fraction workflow described in this study provides a novel methodology to counteract prostate intra-fraction motion without reducing treatment duty cycle and while allowing continuous CINE imaging for motion monitoring. Uniform 2 mm PTV margins are feasible with the described intra-fraction VMAT arc re-optimization strategy.

## Data availability statement

The datasets presented in this article are not readily available because the anonymized datasets used for this research are not currently available for public use. Requests to access the datasets should be directed to jeffrey-snyder@uiowa.edu.

## Ethics statement

The studies involving humans were approved by University of Iowa Institutional Review Board. The studies were conducted in accordance with the local legislation and institutional requirements. The participants provided their written informed consent to participate in this study.

## Author contributions

JS: Conceptualization, Data curation, Formal analysis, Funding acquisition, Investigation, Methodology, Validation, Visualization, Writing – original draft. BS: Software, Writing – review & editing. JSA: Writing – review & editing. AS: Writing – review & editing. DH: Conceptualization, Funding acquisition, Supervision, Writing – review & editing.
